# Mechanism of antibacterial property of micro scale rough surface formed by fine-particle bombarding

**DOI:** 10.1080/14686996.2024.2376522

**Published:** 2024-07-08

**Authors:** Tomoko Nishitani, Takahiko Hirokawa, Hitoshi Ishiguro, Takeshi Ito

**Affiliations:** a Graduate School of Science and Engineering, Kansai University, Suita, Osaka, Japan; b Surf Technology Co. Ltd., Sagamihara, Kanagawa, Japan; c Kanagawa Institute of Industrial Science and Technology, Ebina, Kanagawa, Japan

**Keywords:** Fine particle bombarding (FPB), surface shape, antibacterial effect, oxidative stress

## Abstract

Fine-particle bombardment (FPB) is typically used to modify metal surfaces by bombarding them with fine particles at high speed. FPB is not a coating technique but is used for forming microscale concavities and convexities on a surface. Previously, we reported that an FPB-treated surface showed antibacterial effects; however, the underlying mechanisms remain unclear. We hypothesized that the pitch size of concavity and convexity, and irregular microscale pattern of FPB-treated surfaces might contribute to the antibacterial performance. In this study, we applied FPB to stainless-steel surfaces and evaluated the antibacterial effects of the FPB-treated surfaces based on ISO 22,196:2007. The FPB-treated surfaces exhibited antibacterial activity against *Escherichia coli*, with an antibacterial activity value (R) of two or more. Furthermore, our experiments suggest that the antibacterial mechanism of the FPB-treated surface can be attributed to increased oxidative stress in bacteria owing to physical stress from the rough surface. The antibacterial effect of FPB-treated surfaces offers an effective measure against drug-resistant bacteria.

## IMPACT STATEMENT

The antibacterial activity of FPB-treated surfaces can be attributed to increased oxidative stress induced by physical stress from the FPB-generated rough surface.

## GRAPHICAL ABSTRACT

**Figure:**
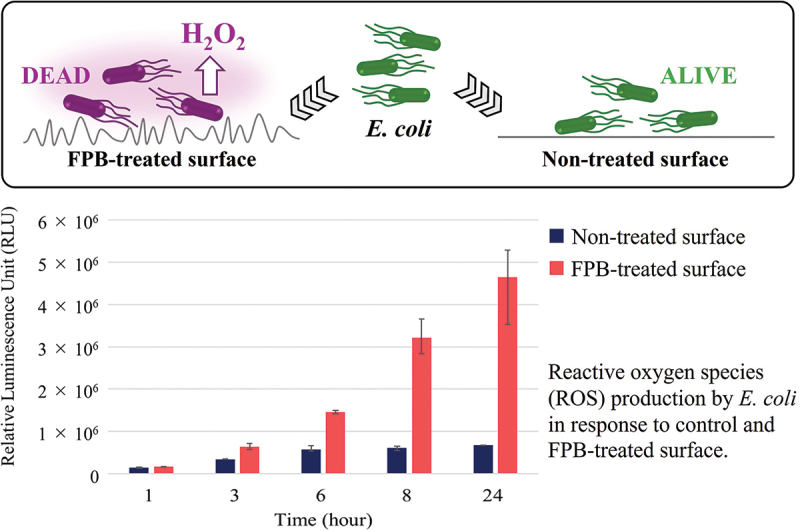

## 1. Introduction

In recent years, the number of deaths caused by drug-resistant bacteria has increased worldwide. The annual death toll from drug-resistant bacteria is 35,000 in the United States and more than 33,000 in Europe [1], and the projected global deaths caused by drug-resistant bacteria is expected to reach 10 million by 2050 [2], exceeding the number of estimated cancer-related deaths. Therefore, materials with antibacterial effects owing to their physical properties and surface shape have attracted considerable attention because they not only combat antimicrobial resistance (AMR) bacteria but also inhibit the proliferation of bacteria. Ivanova et al. reported that nanosized structures on the wings of cicadas and dragonflies exhibit bactericidal activity [3–5]. Subsequently, various artificial nanostructures with antibacterial and bactericidal properties have been actively studied [6–9]. The bactericidal mechanism of nanostructures is considered to include physical disruption of cell membranes, called the stretching effect. In addition, our group has suggested that the antibacterial properties of nanostructures are caused by both physical and biochemical properties, involving the activation of autolytic enzymes after attachment [10].

There has been much research on the antibacterial activity of surface topography, from nanoscale to microscale. Previous studies have reported that microscale rough surfaces can be created by laser processing methods, soft lithography methods, and shot peening methods using relatively large media (120–580 µm) [11–13]. Microscale rough surfaces formed by laser processing and soft lithography methods inhibit bacterial adhesion and biofilm formation.

Fine-particle bombarding (FPB) forms a rough microscale surface by bombarding fine particles, such as ceramics, onto a metal surface (Additional file 1: Figure S1). The size of the medium used is several tens of micrometers or less. One advantage of FPB is its ability to treat large surface areas, up to tens of square meters. Additionally, the fine-particle materials can be reused, limiting the environmental impact, and the processing method or device settings need not be changed for varying shapes of the processed product. This allows for the treatment of complicated three-dimensional shapes. Given the short processing time, ease of handling, and low cost of FPB, microscale asperities can be easily obtained.

In our previous study [14], we confirmed that FPB-treated surfaces with microscale roughness exhibited antibacterial performance against *Escherichia coli* (*E. coli*) and *Staphylococcus aureus* (*S. aureus*). Nanopillar surfaces induced membrane damage in bacteria, whereas FPB-treated surfaces with a strong antibacterial effect were affected by a concavity that matched the size of a bacterium. Scanning electron microscope (SEM) analysis and live/dead assays indicated that *E. coli* on FPB-treated surfaces showed almost no membrane damage, as typically observed on nanostructured surfaces. Therefore, we concluded that the antibacterial effect of the FPB-treated surfaces is not linked to membrane damage. Based on a previous study, we assumed that the antibacterial performance of the FPB-treated surfaces may be related to the physical stress around the microscale roughness, which induces the production of reactive oxygen species (ROS) [15–17].

ROS are produced as a byproduct of normal respiration in mitochondria. When the energy balance or redox balance of bacterial cells is disrupted, the amount of ROS produced increases, and excessive ROS production leads to the damage or death of bacterial cells [18,19]. In bacteria, ROS are primarily generated as byproducts of aerobic respiration [20].

In this study, we used FPB treatment to form microscale rough surfaces and evaluated the relationship between antibacterial activity against *E.coli* and the amount of hydrogen peroxide, a type of ROS that is produced on the FPB-treated surface, as part of the antibacterial mechanism. In addition, we tested the antibacterial property of the FPB-treated surfaces by introducing sodium pyruvate, a reductant for ROS, to the bacterial solution, as pyruvate can reduce hydrogen peroxide.

## 2. Methods

### 2.1. Chemicals and materials

Sodium chloride, 0.1 mol/L sodium hydroxide solution, and 0.1 mol/L hydrochloric acid for salt spray test was purchased from Manac Co. Ltd. (Tokyo, Japan). Nutrient agar (NA) for the ROS assay and nutrient broth (NB) for the antibacterial property tests were purchased from Shimadzu Diagnostics Corporation (Tokyo, Japan). NB for the ROS assay method was purchased from Eiken Chemical Co., Ltd. (Tokyo, Japan) and soybean casein digest broth was purchased from SHIOTANI M.S. Co., Ltd. (Tokyo, Japan). Sodium pyruvate was purchased from Tokyo Chemical Industry Co. Ltd. (Tokyo, Japan). The GloTM H2O2 Assay Kit was purchased from Promega (Madison, Wisconsin, United States). Luminescence was measured using a Glomax Explorer System (Promega, Madison, WI, U.S.A.).

Two types of test pieces were prepared using an SUS304 #700-polished substrate (50 × 50 mm, thickness: 1.0 mm) as a base, which was then subjected to FPB; polished SUS304 #700 was used as a control. The test pieces were labeled as FPB-10 and FPB-40. Substrate was purchased from ZIP MOTOR PRO (Osaka, Japan). The fine particle material used was Densic® (silicon carbide: SiC), with a median particle size of 11.5 ± 1.0 µm for FPB-10 and 30.0 ± 2.0 µm for FPB-40 (Showa Denko K.K., Tokyo, Japan).

### 2.2. Formation of microdimples by FPB

SUS304 stainless steel surfaces with two roughness values were formed using FPB. Surface roughness depends on the composition and size of the fine particles. The conditions for each FPB process are listed in Table 1. The substrate was treated using a BLAST machine (Pneuma Blaster FDQ-2S-L101; Fuji Manufacturing Co., Ltd., Japan). The fine particle materials were mixed with a compressible gas, as shown in Figure S1, and bombarded onto the substrate surface at high speed (150–200 m/s). The nozzle had an inner diameter of 10.5 mm. The effective treatment diameter was about 40–45 mm. Therefore, the sample surface was treated uniformly by moving the nozzle up, down, left, and right. This led to plastic deformation and the formation of irregular and fine asperities, referred to as microdimples, on the treated surface. A laser microscope (VK-X100; KEYENCE, Japan) was used to evaluate the roughness of the FPB-treated surfaces. The roughness parameter of the samples (FPB-10, FPB-40, and the control) were evaluated according to the JIS B0633 protocol. For each group, the number of samples (N) was 3. A scanning electron microscopy (SEM: JSM 7500F, JEOL, Tokyo, Japan) imaging was used to confirm the shape of the FPB-treated surface. Energy dispersive X-ray spectroscopy (EDS: JSM-IT200LA, JEOL, Tokyo, Japan) was used to confirm the Si (atom%) of the fine particle component remaining on the surface.

**Table 1.** Conditions for fine-particle bombardment (FPB).

Test piece	Medium component	Median size (µm)	Air pressure (MPa)	Distance (mm)	Processing time (s)
FPB-10	SiC	11.5 ± 1.0	0.3	150	8
FPB-40		30.0 ± 2.0			5

### 2.3. Salt spray test for FPB-treated surfaces

The polished SUS304 #700 substrate (16 × 37 mm, thickness: 1.0 mm) was used as the control. The corrosion properties of the samples (FPB-10, FPB-40, and the control) were evaluated according to the JIS Z 2371 protocol. We used a spray tester (STP-90 V-2, Suga Testing Machinery, Japan). Exterior photographs of the samples were taken every 24 h without rinsing and drying, and the salt spray test was conducted for 96 h. Photographs of samples after 96 h were taken after rinsing and drying. A 5% sodium chloride solution was used for the test solution; pH was ranged from 6.5 to 7.2 using 0.1 mol/L sodium hydroxide solution and 0.1 mol/L hydrochloric acid solution. The spray volume was 1.5 ± 0.5 mL/80 cm^2^/h. The test temperature was 35°C. The number of each test sample was 1. We measured the corrosion resistance of each test sample by the change in appearance.

### 2.4. Measurement of water contact angle (WCA) on FPB‑treated surfaces

FPB can control the wettability of a treated surface. Reports indicate that the wettability of a surface with a nanopillar structure is related to the viable cell rate [21–23]. In our previous study [14], we found that the lower the WCA, the higher the antibacterial performance, with the number of bacteria adhering to the sample surface increasing with decreasing WCA owing to the hydrophilic cell membrane [24–27]. We evaluated the wettability of the FPB-treated test pieces by measuring their water contact angles (WCAs) using a contact angle meter (DMo-701, Kyowa Interface Science, Japan) with 1.5 μL of purified water droplets. The measurement number of data was 10 for each sample. Thereafter, an antibacterial property test was conducted using the test pieces.

### 2.5. Antibacterial property evaluation

*E. coli* (NBRC3972) was used for antibacterial property tests conducted using FPB-treated pieces and the control as a reference (*N* = 3). The test was performed according to the ISO 22,196:2007 protocol. The antibacterial property tests were conducted under two conditions: (1) a normal antibacterial property test and (2) using a loopful of *E. coli* suspended in a sterile saline solution containing 1.0% w/v sodium pyruvate as the test solution. The *E. coli* concentration was 7.3 × 10^5^ colony forming units (CFU)/mL for the normal test, and 2.7 × 10^5^ CFU/mL for the sodium pyruvate-added test. A test solution (0.4 mL) containing viable bacteria was added to each test piece. Each test piece was covered with a film (Esclinica Pack L, Sekisui Chemical Co., Ltd., Japan) to avoid drying and was maintained at 35°C for 8 h for bacterial growth. The covered film size was 1.6 × 10^3^ mm^2^. Under normal conditions, the test piece was rinsed with a sterile saline solution (9.6 mL). Under sodium pyruvate treatment conditions, the test piece was rinsed using a sterilized saline solution (9.6 mL) containing 1.0% w/v sodium pyruvate. The concentration of the viable bacteria in 10 mL of test solution was estimated using a counting sheet (JNC, Japan). In addition, 1.0% (w/v) sodium pyruvate was added only to the rinsing solution to test its antibacterial properties. The *E. coli* concentration in this test was 1.1 × 10^6^ CFU/mL. Simultaneously, the normal antibacterial property test, without addition of sodium pyruvate to the rinsing solution, was also tested. The other test conditions were identical to those described above.

### 2.6. ROS-GloTM H_2_O_2_ assay method

*E. coli* (NBRC3972) was used for the ROS-Glo^TM^ H_2_O_2_ assay. The method was conducted using FPB-10 and the control as a reference (*N* = 3). *E. coli* cells were grown on NA plates, after which the cells were diluted 1/500 in NB using a sterile inoculation loop to achieve an OD660 of 0.2, where OD660 denotes the optical density of the *E. coli* culture solution at 660 nm. *E. coli* was inoculated into each sample at a volume of 400 µL/sample. Subsequently, the temperature of the test samples was maintained at 35°C with a humidity of at least 90%. The test durations were 1, 3, 6, 8, and 24 h. H_2_O_2_ substrate was added immediately after inoculation at 1, 3, and 6 h. For a test time of 8 h, H_2_O_2_ was added after 2 h of inoculation based on test protocol. For a test time of 24 h, H_2_O_2_ was added 18 h after inoculation. After each test, the test solutions were collected in tubes, and 100 µL of each test solution was dispensed into 96 wells. The ROS-Glo detection solution was added to each well, and the reaction was carried out at room temperature (25 ± 3 ℃) for 20 min, after which the luminescence was measured using a multimode microplate reader (GloMax® Explorer, United State of America).

## 3. Results

### 3.1. Physical properties of FPB-treated surfaces

**Figure 1.**
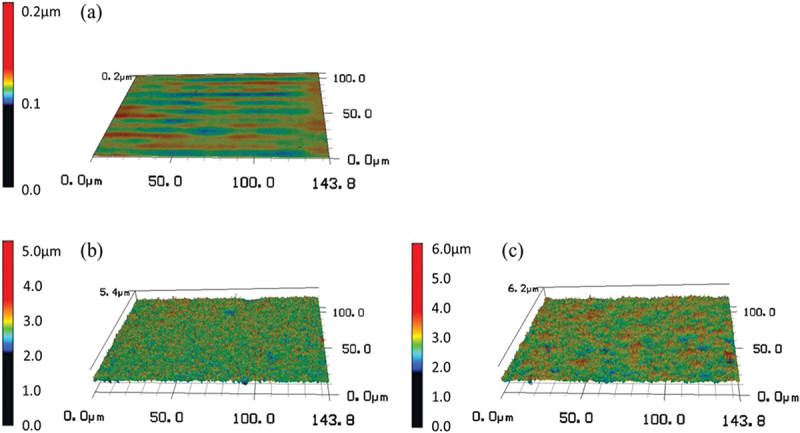
Three-dimensional images of control and fine-particle bombardment (FPB)-treated surfaces. (a) control, (b) FPB-10, and (c) FPB-40.

**Figure 2.**
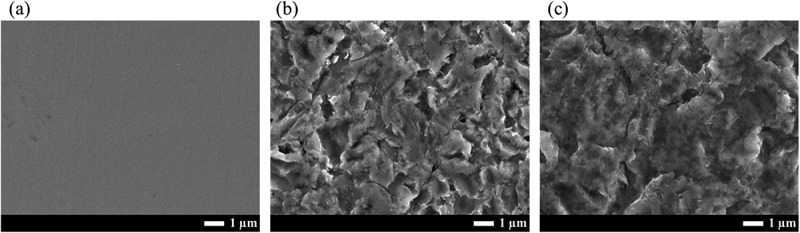
Scanning electron microscope (SEM) images of the control and FPB-treated surfaces (a) control, (b) FPB-10, and (c) FPB-40.

Figure 1 shows the 3D surface images of the control and two different types of FPB-treated surfaces, FPB-10 and FPB-40. Images were obtained using a laser microscope at 2000× magnification. The height scale differed for each test piece. The SUS304 #700-polished surface (control) was smooth, while those treated with FPB-10 and FPB-40 showed concavity and convexity, respectively, with irregular patterns. The roughness was distributed across the entire surface because of the random occurrence of plastic deformation caused by FPB. Figure 2 shows SEM images of the control, FPB-10, and FPB-40 at a magnification of 10,000×. The FPB-treated surfaces show irregular roughness patterns.

Table 2 shows the surface roughness parameters of each sample. In the surface roughness parameter, Rp, Rv, and Rz represent the amplitude parameters of the peak and valley; with Rp being the maximum profile peak height, Rv the maximum profile valley depth, and Rz the maximum profile height. Ra is the average amplitude parameter, which is the arithmetic mean deviation. RSm is the spacing parameter, which denotes the mean width of the profile elements. RΔq is a hybrid parameter, which is the root mean square slope. In summary, the surface roughness parameters, Rp, Rv, Rz, and Ra are parameters that represent the direction of the unevenness height, RSm is a parameter that represents the lateral direction (unevenness pitch) of the unevenness, and RΔq is a parameter that represents the unevenness slope. RSm is applicable (or obtainable) when there are peaks and valleys on the measured surface. As shown in Table 2, the control sample had a smooth surface; therefore, the RSm value was set as a blank. All the roughness parameters were higher for the FPB-treated samples than for the control samples. The median size of fine particles used in the treatment of FPB-40 (30.3 ± 2.0 µm) was larger than that used in the treatment of FPB-10 (11.5 ± 1.0 µm) (Table 1). Therefore, the roughness parameter increased depending on the median size when FPB was used.

**Table 2.** Surface roughness parameters of samples.

Surface Roughness Parameter	Control		FPB-10		FPB-40	
	Range	Average	Range	Average	Range	Average
Rp (μm)	0.004–0.008	0.006	0.294–0.334	0.310	0.497–0.633	0.555
Rv (μm)	0.005–0.007	0.006	0.306–0.370	0.338	0.463–0.754	0.626
Rz (μm)	0.010–0.014	0.012	0.639–0.665	0.648	0.997–1.293	1.181
Ra (μm)	0.002–0.003	0.002	0.080–0.103	0.093	0.156–0.197	0.176
RSm (μm)	–	–	12.386–13.014	12.638	16.048–19.654	18.198
RΔq (°)	0.070–0.080	0.077	6.790–7.160	7.053	8.240–10.680	9.453

Number of measurement samples (N) was 3. The parameter RSm is applicable (or obtainable) when there are peaks and valleys on the measurement surface. Control is a smooth surface (RSm set to blank).

FPB, fine-particle bombardment; Rp, maximum profile peak height; Rv, maximum profile valley depth; Rz, maximum profile height; Ra, average amplitude parameter (arithmetic mean deviation); RSm, spacing parameter (mean width of the profile elements); RΔq, hybrid parameter (root mean square slope).

**Figure 3.**
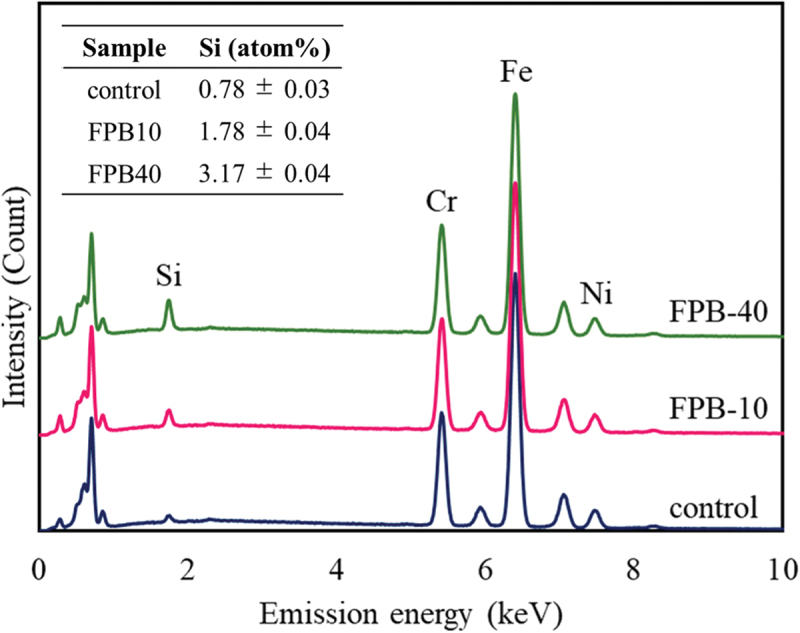
Energy dispersive spectroscopy (EDS) spectra the control and fine-particle bombardment (FPB)-treated surfaces (FPB-10 and FPB-40). The elemental composition ratio of Si (atom%) for each sample is shown in the table in the figure.

The stainless steel used for the test sample was an alloy containing a certain amount of Cr within Fe, making it resistant to corrosion. Metal ions can act as antibacterial agents [28,29]; for example, metallic components of SUS304 such as Cr and Ni may have antibacterial properties with respect to *E. coli* and *S. aureus* [30,31]. However, we confirmed that the antibacterial performance was not dependent on the base material properties, but on the surface shape [32]. Moreover, in the FPB treatment, the bombarded area was repeatedly heated and cooled at a rapid rate. As a result, the treated surface was giving a heat treatment effect. We also confirmed the effect of the fine particles on the surface layer using SEM – EDX analysis (Figure 3). Cr and Ni peaks corresponding to SUS304 was confirmed, with the peak intensities almost unchanged before and after FPB treatment, indicating that the physical properties of the base material were maintained. However, the peak intensity of Si was larger after treatment (FPB-10 > FPB-40 > control), which may reflect a greater number of particles on the surfaces of FPB-10 and FPB-40, or larger particle size. We suggest that the larger particles used to treat FPB-40 were more likely to remain on the substrate surface. No other components were identified on the surfaces and there were no changes in the peak intensities of major elements, indicating that the substrate properties were mostly maintained before and after FPB treatment.

**Figure 4.**
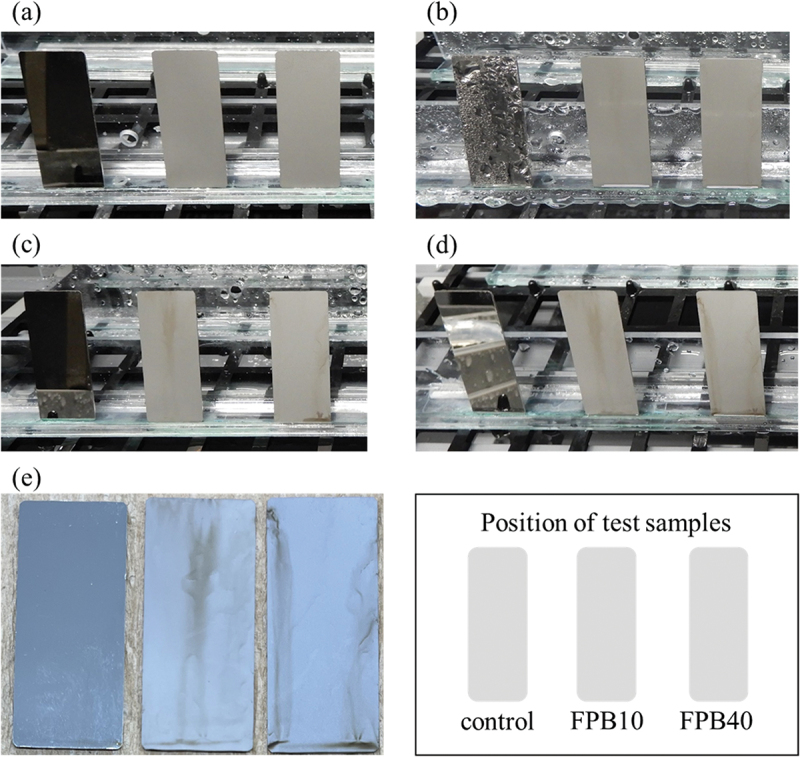
Appearance of samples with salt spray test time. (a) 0 h, (b) 24 h, (c) 48 h, (d) 72 h, and (e) 96 h. Samples position are as shown in the illustration.

**Figure 5.**
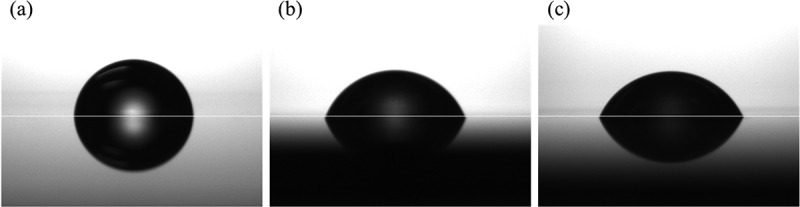
Images for evaluating the water contact angle (WCA) of control and FPB-treated surface. (a) control, WCA = 84.8°; (b) FPB-10, WCA = 70.2°; and (c) FPB-40, WCA = 66.1°.

**Figure 6.**
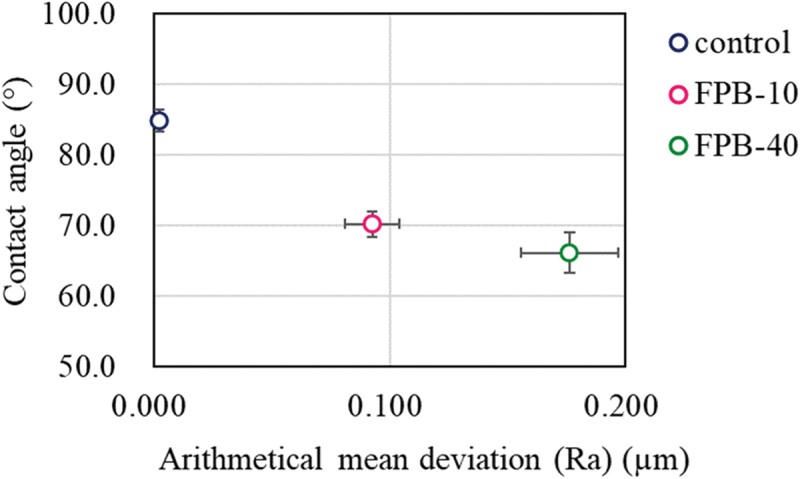
Relationship between the water contact angle (WCA) and Ra for the control and FPB-treated surfaces.

Figure 4 shows exterior photographs of the samples after the salt spray test from 0 to 96 h. FPB-10 and FPB-40 began to discolor from 24 h, with the discoloration clearly visible at 48 h. We attributed this discoloration to rust formation. The images used for evaluating the WCA for each test sample are shown in Figure 5; the numbers shown are the averages of 10 repeat measurements. The WCA values of FPB-10 and FPB-40 were 70.2° and 66.1°, respectively, which are lower than that of the control surface (84.8°). The relationship between WCA and Ra (an amplitude average parameter typically used to represent roughness) are shown in Figure 6. As surface roughness increased, WCA decreased. Therefore, we used good water wettability surfaces as the test samples.

### 3.2. Antibacterial property against E. coli

We tested the antibacterial properties of the FPB-treated surfaces by introducing sodium pyruvate, a reductant for ROS, to the bacterial solution, as pyruvate can reduce hydrogen peroxide, as shown in the reactions below.

$$C_{3}H_{3}\mathrm{Na}O_{3}\to C_{3}H_{3}{O_{3}}^{-}+\;\;\mathrm{Na}\;\;+\;\;C_{3}H_{3}{O_{3}}^{-}+\;\;H_{2}O_{2}\to CH_{3}-\;\;\mathrm{COOH}+CO_{2}+\;\;H_{2}O$$

We considered that a difference in the results of the antibacterial property test using the bacterial solution with or without sodium pyruvate would be indicative of the involvement of hydrogen peroxide in the antibacterial performance of the FPB-treated surface. To confirm that hydrogen peroxide is generated by the test system, the amount of hydrogen peroxide generated per unit test time was also evaluated. The amount of hydrogen peroxide production increased as the number of viable bacteria decreased.

**Figure 7.**
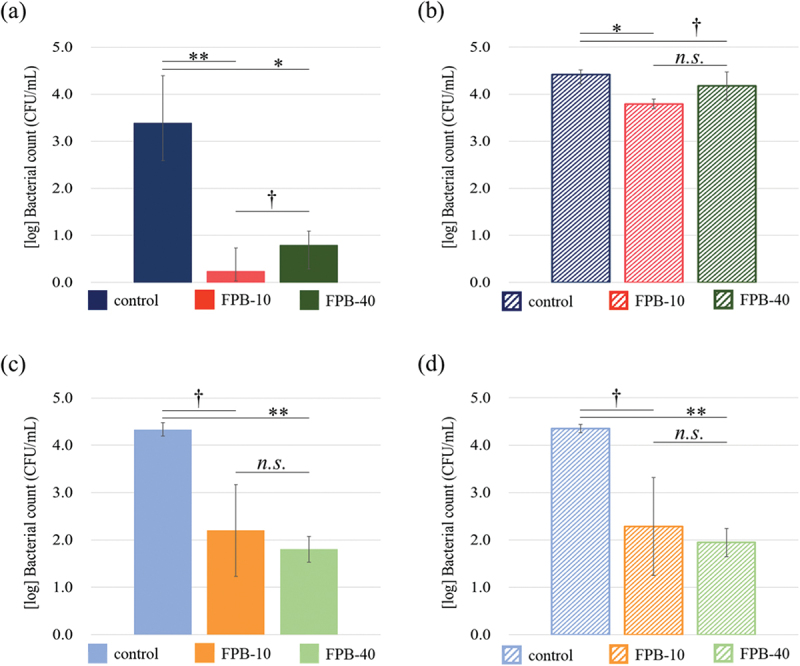
Antibacterial property of fine-particle bombardment (FPB)-treated surface against *E. coli*. (a, c) Normal antibacterial property test with an *E. coli* concentration of (a) 7.3 × 10^5^ CFU/mL and (c) 1.1 × 10^6^ CFU/mL. (b, d) Antibacterial property test using (b) bacterial solution (2.7 × 10^5^ CFU/mL *E. coli* in a sterile saline solution containing 1.0% w/v sodium pyruvate) and rinse-away solution containing 1.0 w/v% sodium pyruvate and (d) bacterial solution (1.1 × 10^6^ CFU/mL *E. coli*) and 1.0 w/v% sodium pyruvate added only to the rinse-away solution. Error bars are standard error.

Figure 7 shows the results of the 8-h tests evaluating antibacterial properties against *E. coli*. In the normal antibacterial property test (Figure 7(a,c)), FPB-10 and FPB-40 showed a reduction in the viable bacterial count by more than two orders of magnitude compared with the control. However, when a bacterial solution containing 1.0% (w/v) sodium pyruvate was used as the test solution for the antibacterial property test (Figure 7(b)), the reduction in the viable bacterial count was in the same range for the control and FPB-treated surfaces. When 1.0% w/v sodium pyruvate was added only to the rinse-away solution (Figure 7(d)), a decrease in the number of bacteria was confirmed for FPB-10 and FPB-40, as in the normal antibacterial property test without the addition of sodium pyruvate. Thus, the timing of the addition of sodium pyruvate produced different results.

### 3.3. Oxidative stress response to E. coli

**Figure 8.**
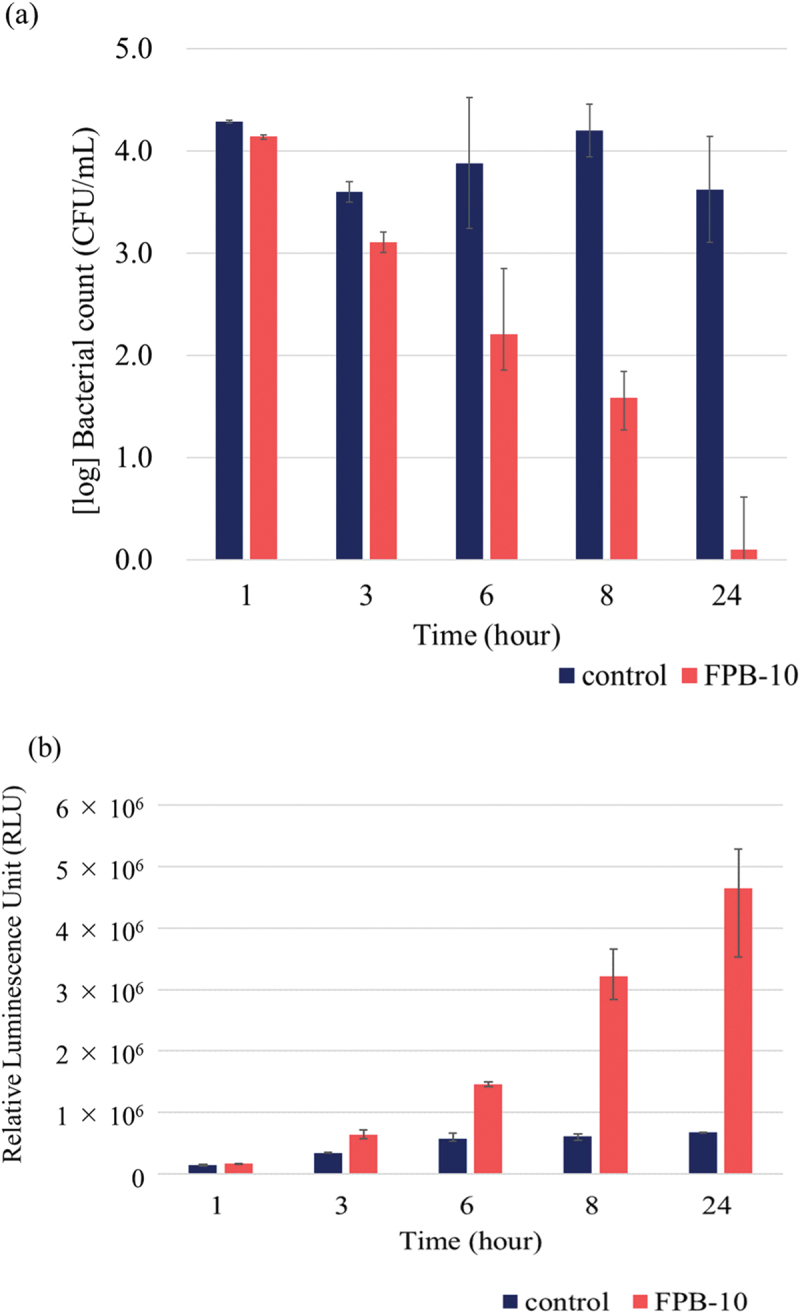
Antibacterial property of FPB-10. (a) Antibacterial evaluation test against *E. coli*. (b) Reactive oxygen species (ROS) production by *E. coli* in response to the control and FPB-10. Error bars are standard error.

Figure 8 shows the results of the antibacterial property test against *E. coli* and the corresponding ROS evaluation tests conducted with control and FPB-10 samples for test durations of 1, 3, 6, 8, and 24 h. The control did not show a significant decrease in the viable bacterial count at any time point, whereas FPB-10 showed a consistent decrease in the viable bacterial count across all evaluated time points (Figure 8(a)). Bacterial reduction of more than two orders of magnitude was observed at 8 h, and a significant reduction was observed at 24 h compared with that in the control. Meanwhile, the ROS evaluation test results showed that the amount of hydrogen peroxide produced increased with time on FPB-10 samples (Figure 8(b)).

**Figure 9.**
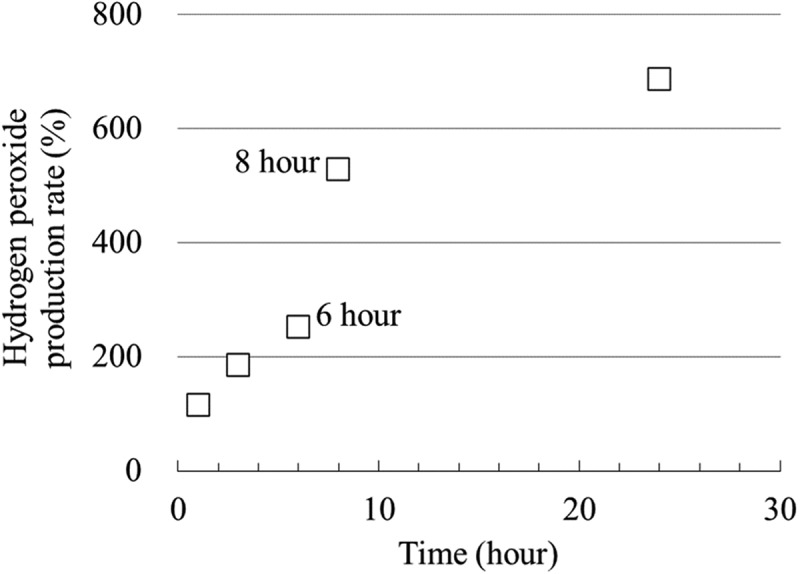
Rate of increase in hydrogen peroxide production per test time on the FPB10-treated surface relative to that on the control surface.

Figure 9 shows the rate of increase in hydrogen peroxide per time point on the FPB-10-treated surface relative to the levels on the control surface based on the luminescence intensity at each time point; the hydrogen peroxide production rate increased with time, particularly from 6 to 8 h.

## 4. Discussion

Comparing the two types of FPB-treated surfaces evaluated in this study, FPB-40 showed higher values for all roughness parameters than did FPB-10. Furthermore, the increase in roughness depended on the median size of the fine particles used. The concavity and convexity of the FPB treated surfaces did not form a regular pattern and the roughness parameters had a range (Table 2). The size of bacteria reportedly changes depending on their life cycle, such as in the logarithmic growth phase [33]. We believe that the irregular pattern shape of FPB-treated surfaces might contribute to antibacterial performance and effectiveness against a bacterial population with a size distribution. According to EDS analysis, fine particles of Si remained on the FPB-treated surfaces. The atomic composition percentages were 0.78 ± 0.03 atom% for the control, 1.78 ± 0.04 atom% for FPB-10, and 3.17 ± 0.04 atom% for FPB-40 (Figure 3). Si reportedly has antibacterial properties [34–36]. Comparing the results of antibacterial evaluation test (Figure 7) with the EDS analysis (Figure 3), we found that the control sample had the highest bacterial count of viable bacteria and lowest atomic composition percentage of Si; there were no significant differences in the antibacterial properties of FPB-10 and FPB-40 (significance level *p* < 0.1 or *p* ≥ 0.1) or between the concentrations of viable bacteria. However, there were significant differences in antibacterial evaluation test results between the control and the FPB-treated surfaces (significance level *p* < 0.01, *p* < 0.05 or *p* < 0.1). Based on these results, we suggest that the antibacterial performance of FPB-treated surfaces is not dependent on the amount of Si, but rather on the surface topology. Moreover, we confirmed that corrosion of the FPB-treated surfaces was high. We suggest that FPB treatment destroys the passive layer on the SUS (e.g. CR_2_O_3_), decreasing the corrosion resistance compared with the non-treated base material.

When sodium pyruvate was added to the test solution and then rinsed off (Figure 7(b)), the bacterial counts were comparable among the control, FPB-10, and FPB-40 groups. In contrast, when sodium pyruvate was added to the rinsing solution (Figure 7(d)), the numbers of bacteria in the FPB-10 and FPB-40 groups were reduced compared with those in the control. *E. coli* ROS production is inhibited by exposure to environmental stresses, such as heating [37,38]. This mechanism is believed to occur owing to damage to the electron transport system localized in the cell membrane. In previous studies, when pyruvate or catalase was added to culture media, damaged bacteria that were originally considered dead showed renewed growth [38–41]. In this study, the addition of sodium pyruvate to the rinsing solution did not sufficiently restore the numbers of *E. coli*. This indicates that *E. coli* on the FPB-treated surface exists in a damaged or dead state and cannot be recovered quickly by the addition of sodium pyruvate to the rinsing solution. This further suggests that ROS is involved in the development of antimicrobial activity on FPB-treated surfaces.

As shown in Figure 8, the number of viable bacteria decreased, whereas the amount of hydrogen peroxide generated increased as the test time elapsed. These results suggest that FPB-treated surfaces exhibit antibacterial activity by inducing an oxidative stress response in *E. coli*. In addition, we confirmed that the bacterial counts did not decrease (Figure 7(b)) when sodium pyruvate was added to the bacteria and the rinsing solution from the beginning. Therefore, we believe that the oxidative stress caused by hydrogen peroxide is involved in the antibacterial mechanism of FPB-treated surfaces. The rate of increase in hydrogen peroxide was significantly elevated from 6 to 8 h (Figure 9). Bacterial adhesion is linked with proteins, water wettability of the material surface, and adsorption of specific molecules. Moreover, bacteria with flagella have an advantage in adhering to rough surfaces [42,43]. We suggested that *E. coli* adhere to FPB-treated surfaces and the oxidative stress induced by keeping adhering on the FPB-treated surface.

The antibacterial property test was conducted in accordance with ISO 22,196:2007, which has an index called the antibacterial activity value (R) that defines the antibacterial performance:

(1) $$R=\log\left(\mathrm{no}.\;\mathrm{viable}\;\mathrm{bacteria}\;\mathrm{after}\;\mathrm{culture}\;\mathrm{on}\;\text{\textbackslash{}breakuntreated}\;\mathrm{test}\;\mathrm{piece}\right)-\log\left(\mathrm{no}.\;\mathrm{viable}\;\mathrm{bacteria}\;\mathrm{after}\;\text{\textbackslash{}break}\mathrm{culture}\;\mathrm{on}\;\mathrm{FPB}-\mathrm{treated}\;\mathrm{test}\;\mathrm{piece}\right)$$

According to ISO 22,196:2007, an antibacterial effect is defined as an antibacterial activity value of 2.0 or higher after 24 h of incubation. In this study, the *R* value exceeded 2.0 after 8 h of testing (Table 3), indicating the presence of an antibacterial effect. In addition, the *R* value increased with time, and a certain level of antibacterial performance was observed even after 6 h. As shown in Figure 8, the oxidative stress response in *E. coli* increased with test time and was highly elevated after 8 h of incubation. When a material surface (in this study, the control and FPB-treated surfaces) is exposed to an *E. coli* bacterial solution, bacterial cells either move in the solution or adhere to the material surface. In this study, compared with the control, the number of viable *E. coli* decreased and the amount of hydrogen peroxide produced increased on the FPB-treated surface. This suggests that FPB-treated surfaces enhance the oxidative stress response when *E. coli* adhere to material surfaces, which inactivates the *E. coli*. Furthermore, the hydrogen peroxide production rate per unit time (Figure 9) indicated a differential hydrogen peroxide production rate, which peaked between 6 and 8 h. This suggests that once *E. coli* adhere to the FPB-treated surface, oxidative stress is gradually induced. In this study, it was not possible to determine how the production of hydrogen peroxide was induced from *E. coli* on FPB-treated surface.

**Table 3.** Antibacterial activity score with time.

Test time (hour)	Antibacterial activity score (R)
1	0.14
3	0.50
6	1.67
8	2.61
24	3.52

The antibacterial performance of FPB-treated surfaces is less immediate than that of antibacterial agents such as ethanol [44], but if the surface morphology is maintained, the effect is semipermanent. Furthermore, the risk of developing AMR bacteria is reduced. Shot peening is a surface modification treatment method that is similar to FPB, but with larger particle sizes. However, although the surface formed by shot peening reduces bacterial adhesion, it had a lower antibacterial effect than that observed in our study [11]. This could be attributed to the difference in the roughness of the pitch. In conclusion, we believe that FPB-treated surfaces offer a potential countermeasure against the global issue of AMR bacteria.

## Supplementary Material

Supplemental Material

## Data Availability

The datasets analyzed during the current study are available from the corresponding author upon reasonable request.
