# Supplementary figures and images for: Mechanism of antibacterial property of micro scale rough surface formed by fine-particle bombarding
Source: Sci Technol Adv Mater. 2024 Jul 8;25(1):2376522. doi: 10.1080/14686996.2024.2376522 (PMC11271079; doi:10.1080/14686996.2024.2376522)

## Slide 1
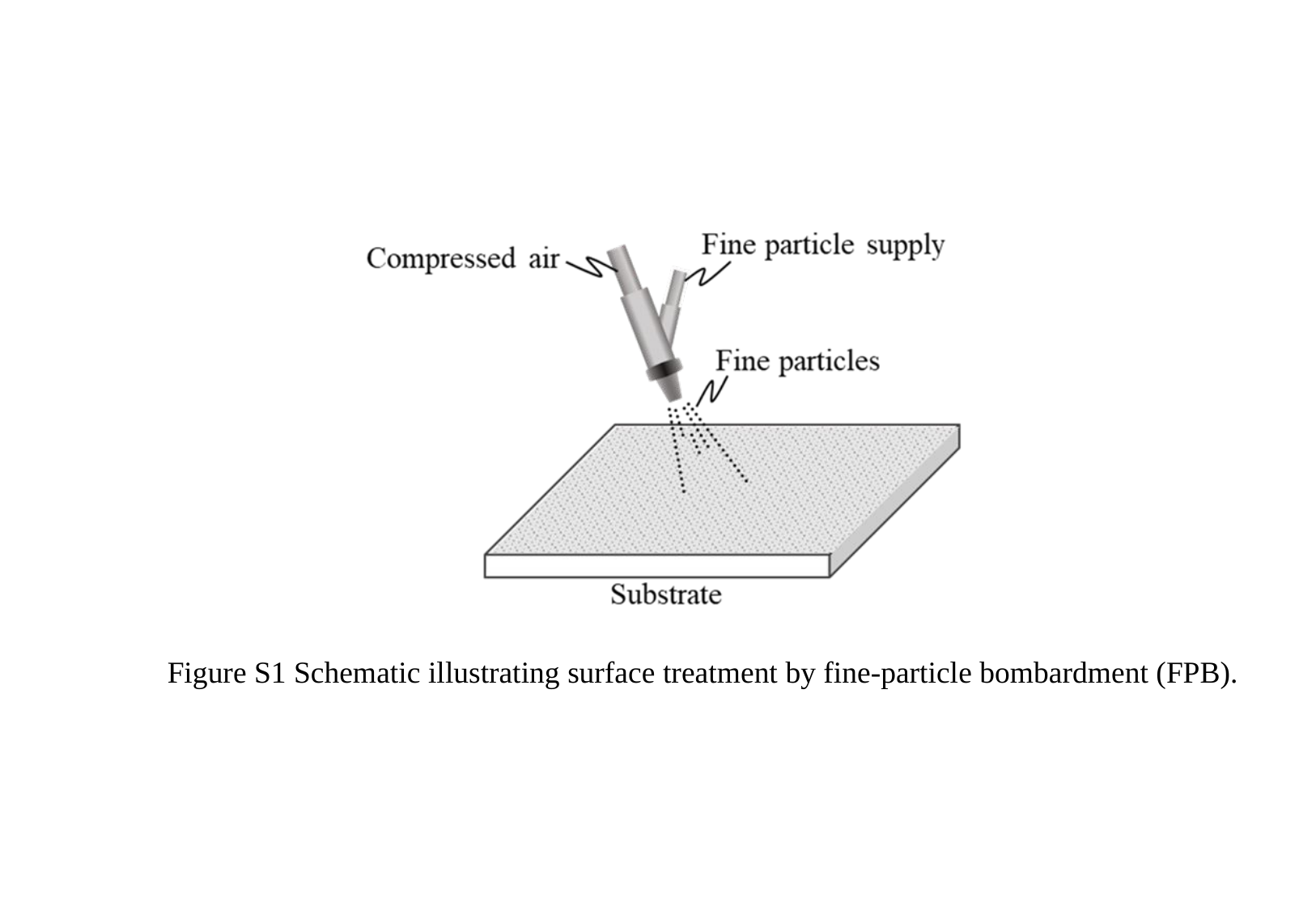

Figure S1 Schematic illustrating surface treatment by fine-particle bombardment (FPB).

Supplement: Supplemental Material [file TSTA_A_2376522_SM8999.pptx]
